# Preparation of nanoliposomes by microfluidic mixing in herring-bone channel and the role of membrane fluidity in liposomes formation

**DOI:** 10.1038/s41598-020-62500-2

**Published:** 2020-03-27

**Authors:** Jan Kotouček, František Hubatka, Josef Mašek, Pavel Kulich, Kamila Velínská, Jaroslava Bezděková, Martina Fojtíková, Eliška Bartheldyová, Andrea Tomečková, Jana Stráská, Dominik Hrebík, Stuart Macaulay, Irena Kratochvílová, Milan Raška, Jaroslav Turánek

**Affiliations:** 1 Department of Pharmacology and Immunotherapy, Veterinary Research Institute, v.v.i., Hudcova 70, 621 00 Brno, Czech Republic; 2 Mendel University in Brno, Department of Chemistry and Biochemistry, Zemedelska 1, 61300 Brno, Czech Republic; 3 Regional Centre of Advanced Technologies and Materials, Palacký University, Šlechtitelů 11, 78371 Olomouc, Czech Republic; 4 Central European Institute of Technology CEITEC, Structural Virology, Masaryk University, Kamenice 753/5, 62500 Brno, Czech Republic; 5 Malvern Panalytical, Malvern, Worcestershire, United Kingdom; 6 Institute of Physics, Czech Academy of Sciences, Na Slovance 2, Prague 8, Czechia; 7 Department of Immunology, Faculty of Medicine and Dentistry, Palacky University Olomouc, Hněvotínská 3, 775 15 Olomouc, Czech Republic

**Keywords:** Biomaterials, Nanoscale materials

## Abstract

Introduction of microfluidic mixing technique opens a new door for preparation of the liposomes and lipid-based nanoparticles by on-chip technologies that are applicable in a laboratory and industrial scale. This study demonstrates the role of phospholipid bilayer fragment as the key intermediate in the mechanism of liposome formation by microfluidic mixing in the channel with “herring-bone” geometry used with the instrument NanoAssemblr. The fluidity of the lipid bilayer expressed as fluorescence anisotropy of the probe N,N,N-Trimethyl-4-(6-phenyl-1,3,5-hexatrien-1-yl) was found to be the basic parameter affecting the final size of formed liposomes prepared by microfluidic mixing of an ethanol solution of lipids and water phase. Both saturated and unsaturated lipids together with various content of cholesterol were used for liposome preparation and it was demonstrated, that an increase in fluidity results in a decrease of liposome size as analyzed by DLS. Gadolinium chelating lipids were used to visualize the fine structure of liposomes and bilayer fragments by CryoTEM. Experimental data and theoretical calculations are in good accordance with the theory of lipid disc micelle vesiculation.

## Introduction

Liposomes, self-assembled nanoparticles based on lipid bilayers are widely employed for biomedical and biotechnological purposes^1^. Liposomes are composed of one or more lamellae, consisting of a phospholipid bilayer and enclosing a small volume of aqueous liquid. The diameter of liposomes can vary from tens of nanometers up to hundreds of micrometers depending on the method used for their preparation. Unilamellar or oligolamellar liposomes with an average size of about 80 nm are typically produced and used in medical applications as anticancer drug delivery systems^2^.

Phospholipids and lipids, the main components of liposomal membranes, are well soluble in various organic solvents, therefore most present methods use organic solvents (e.g. methanol, ethanol, terc-butanol, chloroform, ethers) to solubilize lipids as a first step in the whole procedure. If the organic solvent is miscible with water, liposomes can be prepared by mixing an alcoholic solution of lipids with aqueous phase. Ethanol injection method^2^ and proliposome-liposome method^2,3^ represent well-established techniques used in the laboratory as well as on an industrial scale^4–6^.

The advent of microfluidics opens a new door for the on-chip preparation of liposomes and other lipid-based nanoparticles^7^. Microfluidic methods have demonstrated their ability to control the process of mixing the organic and water phases, therefore the required parameters of final liposomal products like size, polydispersity, morphology, and lamellarity are achieved in a more reproducible way.

Various systems were designed and constructed for laboratory use and also for the application of this technology on an industrial scale. Microfluidic approaches to liposome production are based on the application of various geometries of mixing channel as well as the application of shear forces, electric field or various microfluidics effects taking place in the channel. A comprehensive review was published recently by Carugo *et al*.^8^, Wang *et al*.^9^ and Maeki^10,11^. Theoretical models describing kinetic and thermodynamic parameters that determine the formation of liposomes from lipid bilayer disc were described^12–15^. The process of microfluidic mixing can help in optimizing the method, especially for the production in industrial scale using complex on-chip technology as recently demonstrated^16,17^.

Here we used the microfluidic mixing channel with “herring bone” geometry to perform nanoprecipitation within milliseconds and nanoliter reaction volumes. “Herring bone” geometry of mixing channel differentiates this process from microfluidic mixing based on microfluidic hydrodynamic flow focusing of an alcohol stream with two aqueous buffer streams^18^.

**Figure 1:**
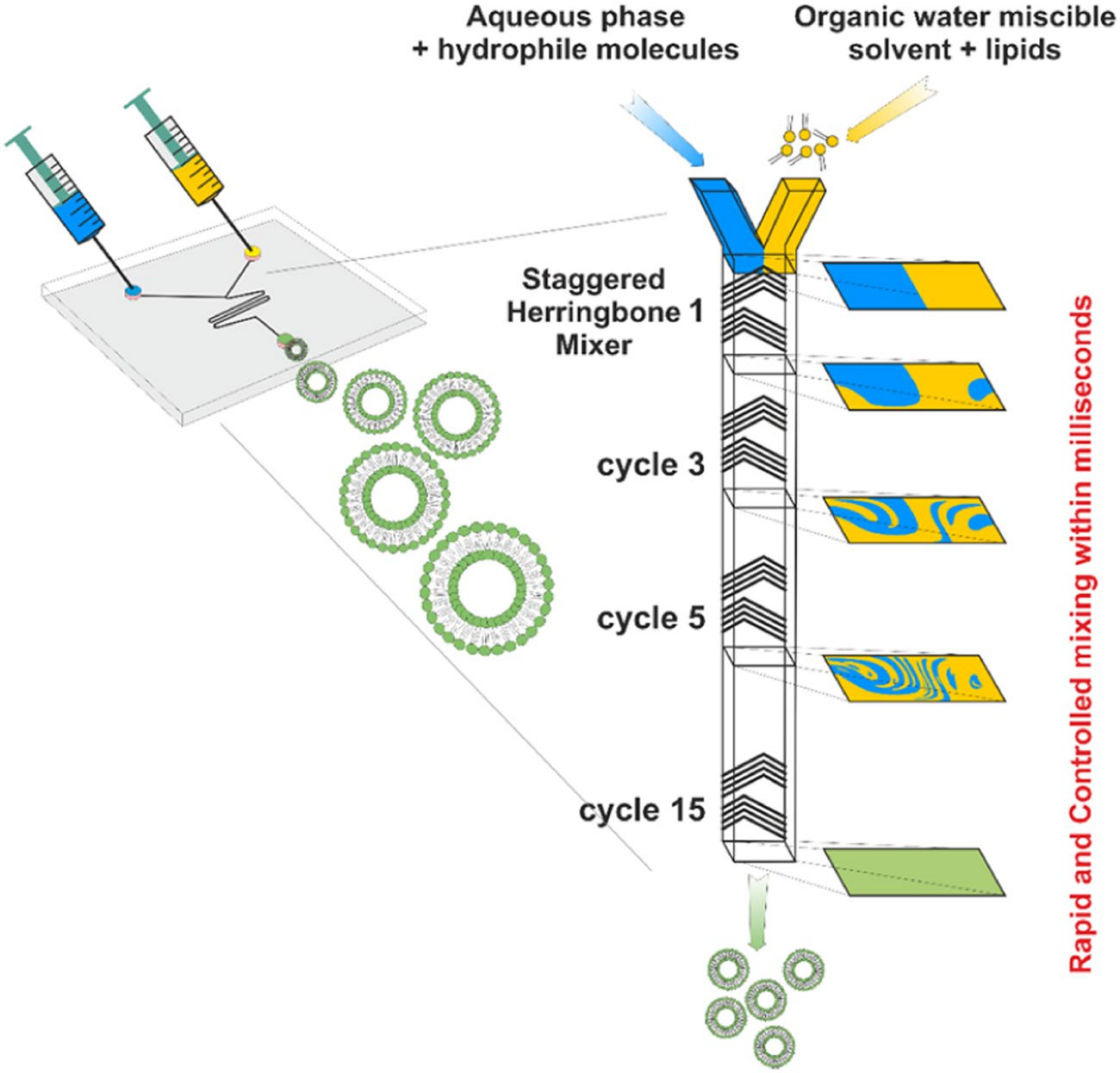
Principle of microfluidic mixing in herring bone channel and formation of liposomes. Organic water miscible solvent (ethanol) contains lipids forming liposomes while water phase contains water soluble components that are to be encapsulated. The mixing process is finished within millisecond and liposomes are formed by the self-assembled mechanism. Various linear injector syringe pumps controlled by computer are used to drive the mixing process.

“Herring bone” geometry mixing channels are now available as commercial products together with computer-controlled instruments for laboratory and industrial scale preparation of lipid and polymeric nanoparticles. Therefore, the method is available for wide use to prepare nanoliposomes. The schematic illustration of the preparation of liposomes by microfluidic mixing in herring bone chamber is presented in Fig. 1.

In this study, we applied gadolinium-chelating lipids and cryo-electron transmission microscopy to visualize in detail the structure of liposomes, liposomal membrane, and intermediates formed within the whole process. We tested the effect of lipid membrane fluidity on the final morphology of liposomes expressed as the size distribution, polydispersity, and lamellarity. Based on exact measurements of lipid membrane fluidity (polarization of fluorescence) we predicted the effect of this parameter on the critical size of disc micelle intermediates and therefore their tendency to vesiculate and form liposomes^3,19,20^. Experimental data were compared with theoretical prediction following from the model based on Helfrich bending energy and analyses of the energetics and thermodynamics of vesicle formation^15^.

## Results

### The size distribution of liposomes composed saturated and unsaturated lipids

In the first experiment, the prediction based on the theoretical model describing the critical size of liposomes composed of various lipids was verified^15^. The sizes of liposomes composed of EPC, SOPC, and DMPC were compared to published theoretical and experimental data^21,22^. The size distribution of liposomes prepared by microfluidic mixing were in a good accordance with the theoretical prediction. The results are summarized in Table 1. We were not able to prepare liposomes composed only form DSPC without the addition of cholesterol. Therefore, the data on pure DSPC were not included in Table 1 and also in distribution analyses.

**Table 1:** Comparison of liposomal theoretical diameter with sonicated vesicles and vesicles prepared by microfluidic mixing.

Lipid type	*D*_min, calculated_ # (nm)	*D*_sonication_ # (nm)	*D*_min, calculated *_ (nm)	*D*_microfluidic_ (nm)
EPC	17.3	21.8	ND	22.0
DMPC	13.4	16.8	14.1	14.3
SOPC	ND	ND	17.1	22.3

From the number distribution (D_microfluidic_) the minimal size of the vesicles was compared with theoretical values (D_min, calculated_), vesicles base on the sonication method (D_sonication_).

^#^Data from citation^15^.

^*^D_min_ calculation based on d_0_ and d published in Handbook of lipid bilayers, second edition, Derek Marsh^33^.

### Effect of cholesterol on the size distribution of liposomes prepared by microfluidic mixing

Liposomes prepared by microfluidic mixing exhibit various size properties based on the saturated/unsaturated chains of lipids and on the cholesterol content while maintaining the same preparation conditions as the Flow Rate Ratios (3:1) and Total Flow Rate (7 ml/min).

By Flow Rate Ratios (FRR) is meant the volumetric ratio of the organic and aqueous medium mixed through the microfluidic channel and Total Flow Rate is defined as a sum of the volumetric ratio of organic and aqueous medium pumped through the two inlets.

### Preparation of liposomes composed of unsaturated lipids

**Figure 2:**
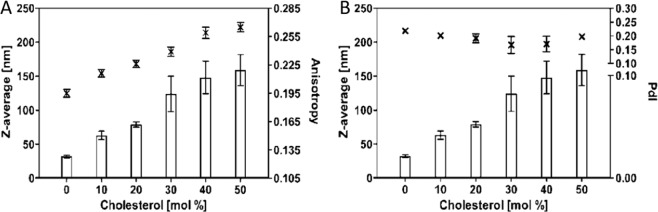
Effect of cholesterol on size, polydispersity, and anisotropy of liposomes prepared from unsaturated phospholipids (EPC). (**A**) The plot of cholesterol concentration (mol %) versus Z-average diameter (nm) on the major axis and steady state fluorescence anisotropy of the DPH-TMA on the minor axis. (**B**) The plot of cholesterol concentration (mol%) versus Z-average diameter (nm) on the major axis and polydispersity index (PdI) and on the minor axis.

For unsaturated lipids as EPC, the increase of cholesterol content resulted in an increase in the size of the liposomes (Fig. 2). Increased cholesterol content in the lipid bilayer composed of unsaturated phospholipids resulted in a decrease in the fluidity/elasticity of liposomal lipid bilayer. This effect was demonstrated by both complementary physical-chemical parameters as an increase of anisotropy and a decrease of fluidity which is directly related to the elasticity of the phospholipid bilayer. Fluidity was expressed as anisotropy of steady state fluorescence of molecular probe DPH-TMA. Steady-state anisotropy of the probe in non-organized systems such as in n-heptane reached values 0.001. In organized systems such as liposomes, DPH-TMA is oriented along the hydrocarbon chains^20^ in which rotation is reduced and anisotropy is increased. With decreasing fluidity of the bilayer structure, anisotropy values increased from 0.195 (without cholesterol) to 0.265 (50 mol% of cholesterol) (Fig. 2). PDI of prepared liposomes was not substantially changed by cholesterol content and the PDI values were within the range 0.17–0.23.

### Preparation of liposomes composed of saturated lipids

**Figure 3:**
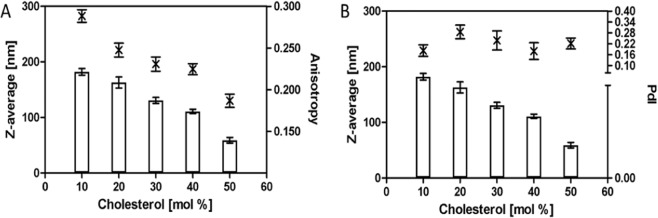
Effect of cholesterol on size, polydispersity, and anisotropy of liposomes prepared from saturated phospholipids (DSPC). (**A**) The plot of cholesterol concentration (mol %) versus Z-average diameter (nm) on the major axis and steady state fluorescence anisotropy of the DPH-TMA on the minor axis. (**B**) The plot of cholesterol concentration (mol %) versus Z-average diameter (nm) on the major axis and polydispersity index (PdI) and on the minor axis.

Saturated lipids such as DSPC exhibit different properties. Cholesterol in bilayer structure is increasing the fluidity of the fragment which allows the formation of smaller vesicles (Fig. 3). Steady state anisotropy is gradually decreasing with increasing cholesterol content from 0.288 to 0.187 for 10 mol% and 50 mol% respectively. In comparison to unsaturated phospholipids, PDIs of liposomes composed of saturated phospholipids were slightly higher, but again they were not substantially changed by cholesterol content and were within the range 0.20–0.28.

**Figure 4:**
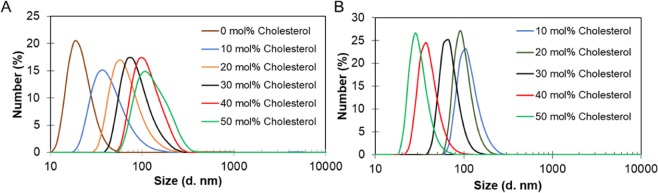
Size distribution by the number of (**A**) EPC/Cholesterol and (**B**) DSPC/Cholesterol liposomes with increasing cholesterol concentration from 10 mol% to 50 mol%.

**Figure 5:**
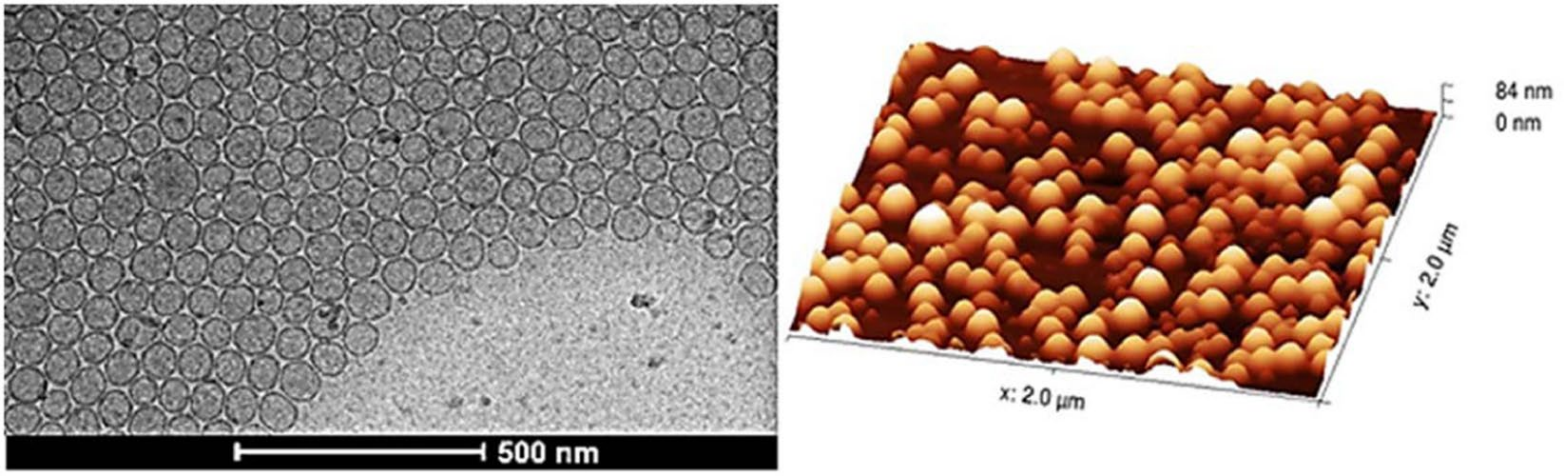
Structure of liposomes revealed by cryoelectron transmission microscopy and atomic force microscopy. Liposomes were prepared by the microfluidic mixing method. The composition was DSPC:Chol 70:30 Cryo-TEM picture (left) and AFM picture (right).

Size distribution by number reflects the most numerous population of liposomes. The effect of cholesterol on the size distribution of liposomes composed of unsaturated and saturated phospholipids is presented in Fig. 4. The example of liposomes (DSPC:Chol 70:30) prepared by microfluidic mixing and visualized by cryoTEM and AFM is in Fig. 5. Unilamellarity of liposomes and homogeneity in the size distribution is in correlation with DLS data.

### Electron microscopy of Gd-labelled liposomes

Phosphorus atoms in phospholipids are responsible for contrast in cryo-electron microscopy enabling visualization of the phospholipid bilayer. Atoms of gadolinium chelated in Ga-lipids represent much more dense material for electrons, therefore sharp and strong visualization of the lipid bilayer in liposomes can be achieved.

**Figure 6:**
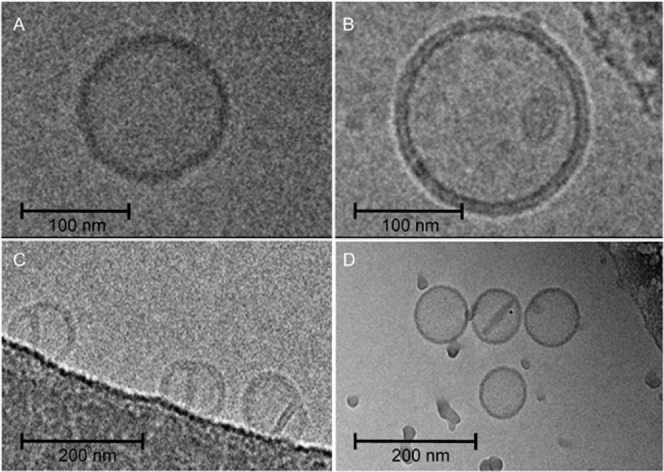
Cryo-TEM images of a liposomes: (**A**) control sample (Table 1, composition 3), width of phospholipid bilayer ≈ 4.3 nm, (**B**) liposomes containing 5% of Gd-lipid (Table 1, composition 3), width of phospholipid bilayer ≈ 5.8 nm, (**C**) liposomes (Table 1, composition 1) with a lipid membrane fragment and (**D**) conjugated liposomes (Table 1, composition 3).

The resolution of the outer and inner lipid layers of the liposomal bilayer can be clearly observed because of the larger distance of Gd in the bilayer when compared to phosphorus atoms (Fig. 6A,B). Therefore, the bilayer character of fragments of bilayers (Fig. 6D), as well as a bilayer partition separating two conjugated liposomes (Fig. 6C), were demonstrated by cryo-electron transmission microscopy. The presence of Gd in liposomes was confirmed by TEM-EDX (Supporting data Fig. S1A,B) and three Gd peaks were identified when liposome was scanned (Supporting data Fig. S1B).

## Discussion

Microfluidic mixing represents a promising technology for the production of complex liposomal preparations^15^ and the application of on-chip technology as demonstrated recently^3,10^. Gadolinium complexes are used as MRI contrast agents and liposomes represents a versatile platform for the application of gadolinium *in vivo* imaging and development of theranostics^23–25^. Gadolinium lipid complexes represent also a useful tool for visualization of lipid structures by cryoTEM as we demonstrated in this article.

Knowledge of mechanisms of liposome formation during microfluidic mixing is of importance to optimize the process on both laboratory and industrial scale. Various forms of bilayer phospholipid fragments (BPF) were supposed as intermediates during the process of liposome formation^26^. For example, disc-like bilayer micelles (DBM) are intermediates formed during the process of the detergent removal method and vesiculation of these disc micelles creates liposomes very homogeneous in their size distribution^27,28^. On the other hand, stacked bilayer fragments are the main intermediates formed in the proliposome-liposome method^12,13^. Because the liposome preparation by application of microfluidic mixing method is based on similar principles, such as the proliposome-liposome method or ethanol injection method, it is reasonable to assume that BPF in the form of DBM is also an intermediate in the process of liposome formation.

Vesiculation of bilayer disc micelles in aqueous milieu is a spontaneous process driven by the minimization of the line tension energy originated in the exposure of the nonpolar hydrophobic tails of lipid molecules, which are presented along the edge of the disc membrane micelles, to polar water molecules. Vesiculation, as the process of transformation of a planar bilayer membrane into a sphere, is hindered by the resistance of the lipid membrane towards bending. Therefore, it becomes possible only when the driving force is sufficiently high to overcome the corresponding resistance towards bending.

**Figure 7:**
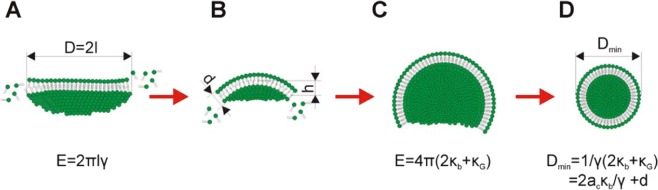
A schematic illustration of vesicle formation in the process of self-assembly. A planar disc-like micelle (**A**) curves into a spherical cap (**B** and **C**) and finally closes to form a vesicle (**D**). Equations are described in the text.

The formation of vesicles starts within milliseconds from small aggregates of individual amphiphilic lipid molecules to disk-like bilayer structures formed in the rapid process of self-assembly. (Fig. 7A). Growing bilayer structures generally tend to maintain a circular disc configuration to keep the overall line tension energy at a minimum. After reaching a certain critical size the bilayer structure tends to curve to further reduce the line energy. (Fig. 7B,C). Finally, the cup-like structure closes to form a spherical vesicle, as shown in Fig. 7D. This mechanism implies the formation of a vesicle of minimal size related to the minimal size of disc micelle.

The reduction in line energy during the transition from a planar DBM to a closed vesicle is countered by an increase in the membrane bending energy. The size of vesicles depends on membrane size growth kinetics, but there is a critical membrane size below which vesiculation is energetically unfavorable. It means that below the critical diameter of DMB vesicles are not formed and it implies also a critical diameter of vesicles, which depends on lipid composition determining the fluidity/flexibility of lipid bilayer.

Basic equations describing the energy of various intermediates in the process of liposome formation was recently described by Huang *et al*.^15^. Planar disc bilayer micelle with a diameter of *D* = 2 *l*, as shown in Fig. 7, was considered by the authors and they described the system energy by the equation:

1 $$E=\frac{1}{2}\pi {k}_{b}\cdot {\left(\frac{4h}{l}-l{c}_{0}\right)}^{2}+4\pi {k}_{G}\cdot {\left(\frac{h}{l}\right)}^{2}+\frac{16}{{l}^{2}}\pi B\cdot {\left(\frac{h}{l}\right)}^{4}2\pi \sqrt{{l}^{2}-{h}^{2}\gamma }$$

where *h* ∈ [0, *l*] is the height of the spherical cap that effectively indicates vesicle shape evolution (*h* = 0) corresponds to a planar patch; *h* = *l* corresponds to a spherical vesicle, *B* = *κ*_1_ + *κ*_2_ + *κ*_3_, where *κ*_1_, *κ*_2_, and *κ*_3_ are fourth-order moduli; *γ* is line tension; *κ*_G_ is Gaussian bending stiffness, *κ*_b_ is the bending stiffness, and *c*_o_ is the spontaneous curvature. The last term in Eq. (1) is the line energy along the free edge, where *γ* is the line tension.

Based on assumption that the number of lipid molecules in the micelle and formed liposome remains constant (it implies the same surface of micelle and vesicle formed from it), during the process of vesiculation, the membrane is assumed to maintain a uniform curvature. For an initially flat disc-liker membrane micelle *h* = 0 and the system energy in Eq. (1) reduces to *E*_1_ = 2*πlγ*. For a fully vesiculated sphere *h* = *l* and Eq. (1) is reduced to *E*_2_ = *8πκ*_*b*_ + *4πκ*_*G*_ + *16πB/l*^2^, which can be further reduced to *E*_*2*_ = *4πκ*_*b*_ for the case of linear elasticity *B* = 0 and *κ*_*G*_ ≈ *−κ*_*b*_^29^_._

For the transition from the DBM configuration into a spherical vesicle, it must stand that E_2_ ≤ E_1_. Based on above assumption the lower diameter of the circular DBM that can possibly form a spherical vesicle is given by l^*^ *= 2κ*_*b*_*/γ, l* = al^*^, where the scaling parameter *a* ≥ 1^15^.

The lower outer diameter of spontaneously self-assembled vesicles can be expressed as:

2 $${D}_{\min }={a}_{c}{l}^{\ast }+d=2{a}_{c}\frac{{k}_{b}}{\gamma }+d$$

where *d* is the thickness of the membrane bilayer and a critical membrane size *l* = *a*_*c*_l^*^, where *a*_*c*_ is the critical scaling parameter. Because differences in the thickness of the membrane formed from different phospholipids are relatively small, the main factor effecting *D*_*mi*n_ is the value of ***κ***_***b***_. While **κ**_**b**_ is related to the elasticity of the membrane reflected also by its fluidity, parameter **γ** depends presumably on the character of hydrocarbon chain (e.g. content of saturated and unsaturated acyls in phospholipid molecule) and its length. Therefore, the critical diameter of the disc micelle l* is strongly dependent of **κ**_**b**_ as demonstrated by the effect of cholesterol on the size of liposomes (Figs. 1–3). Bending energy determines the critical size of DBM and is the crucial thermodynamic parameter ruling the process of vesiculation of DBM. Lipid composition of DBM and temperature are the main factors effecting bending energy and therefore the final size of formed vesicles. Direct mathematical relation of bending stiffness (generally obtained from measurements on giant liposomes), linear tension and anisotropy are not described by theoretical models. We used fluorescence anisotropy data as a parameter reflecting the relative fluidity of phospholipid bilayers with the different cholesterol content. Parameters like fluidity/elasticity of the lipid bilayer reflect bending energy can be obtained experimentally by independent methods e.g. measurement of anisotropy of fluorescence, as we applied in this study.

During the process of microfluidic mixing of water and alcohol-lipid phases, the edges of DBM are not stabilized and any formed DBMs are short lived metastable structures. The thermodynamic instability at the edges of the DBM causes bending and when the DBM closes upon itself, a vesicle is formed. Therefore, the vesiculation of DBM is driven by minimizing their edge energy. As the bilayer rearranges from a flat disc into a sphere, the total energy of the system first increases due to contributions from the bending energy of the bilayer. Subsequently, the total energy decreases as the edges disappear during the process of closing of DBM and finally disappear when vesicles are formed.

Fluidity (reflected by the value of anisotropy) correlated well with the final size of liposomes (Figs. 1–3) and their lipid composition. The solidification effect of cholesterol on lipid membranes composed of unsaturated phospholipids lipids (in our study we used EPC) was reflected by a decrease of the fluidity of the membrane. The size of the liposomes was increased as predicted by the model based on DBM. An opposite effect of cholesterol was observed on membranes formed by saturated phospholipids (in our study we use DPPC). The softening of the phospholipid membrane was reflected by an increase of fluidity. The final size distribution of liposomes increased with increasing cholesterol content in the membrane. The role of fluidity as the crucial parameter ruling the vesiculation of DBM is supported by the comparison of the values of anisotropy and the size of liposomes prepared from unsaturated or saturated lipids mixed with cholesterol (compare Figs. 1 and 2).

Nevertheless, present models of liposome formation are static while the formation of disc micelles and their vesiculation are highly dynamic processes. Therefore, kinetic aspects of vesicle formation are responsible for an increase of the final size of liposomes above theoretical values, predicted from static models. Moreover, static models do not involve the important factors such as shearing forces occurring during rapid flow mixing in the herring-bone like channel.

Formation of larger vesicles can be eliminated by shearing forces and septum in coalescent liposomes can be forced apart of the bilayer to form disc micelles encapsulated inside the liposomes (Fig. 6C,D), as discussed below. One has to keep in mind, that the DLS method gives an average size of liposomes, while the critical size of liposomes (smallest liposomes) can be measured only by cryoTEM.

**Figure 8:**
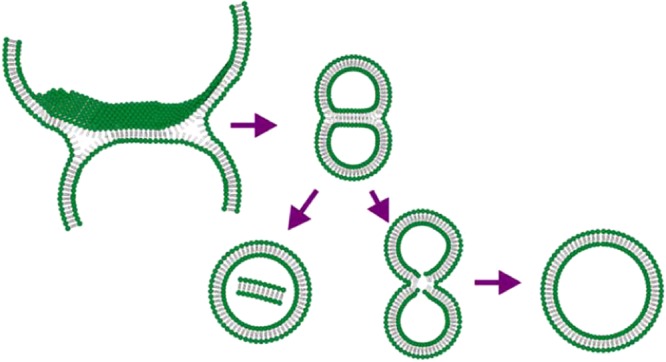
Schematic description of possible rare transition intermediates in the process of liposome formation by microfluidic mixing.

Jahn and colleagues used cryo-SEM methods to visualize lipid structures formed during the process based on microfluidic hydrodynamic flow focusing on an alcohol stream with two aqueous buffers streams^19^. In this study, the authors used cryo-SEM imaging to demonstrate a mixture of spherical vesicles as well as disc-like lipid structures, fractured or incomplete vesicles, and flat aggregate structures. The disc-like bilayer fragments were presented in rapidly frozen samples observed by cryo-SEM. Application of the above-mentioned method is technically difficult if impossible for a herring bone like channel in the compact cartridge. Therefore, we used the approach based on cryo-TEM and Gd-chelating lipids to search for residual traces of various intermediates and to visualize a detailed structure of the lipid double layer in liposomal membranes. Firstly, we confirmed the presence of Gd in the liposomal bilayer by TEM with EDX to rule out possible misinterpretations. The spatial co-localization of Gd with liposomes was proved by this method. Therefore, the clear resolution of both lipid layers forming a liposomal bilayer can be attributed to Gd providing strong electron contrast (Fig. 4). Among many perfectly formed liposomes, we were able to find several liposomes preserving imperfections pointing to intermediates formed during the process of liposome formation. In Fig. 6C we can see conjugated liposomes with a bilayer septum dividing their internal space. One liposome with a disc-like bilayer fragment inside the internal volume is presented (Fig. 6D). These two morphological structures point to the mechanism of liposome formation based on the assembly of DBMs and their vesiculation when their critical size is reached. The proposed mechanism is described in Fig. 8.

The Fig. 8 indicates that morphological and size distribution uniformity of liposomal preparations produced by the microfluidic mixing method is ruled by the physical-chemical character of DBM, which can be expressed in well-defined and quantifiable terms as the fluidity of the lipid double-layer membrane.

Application of Gd-lipids and cryoTEM revealed some residual “witness” of bilayer structures formed in the process of microfluidic mixing. BPF is first formed in the early stages of mixing. This was also supposed by Maeki and colleagues who studied the effect of flow rate on the size of POPC liposomes prepared by microfluidic mixing in herring-bone channel^11^.

The mechanism of vesiculation is the reason for the high uniformity of liposomal preparations obtained by the method of microfluidic mixing. Of course, at higher concentration of lipids the rapid growth of DBM can lead to the formation of imperfection in DBMs like forks on their rims and finally coalescent liposomes are formed. These rare liposomal structures can be stabilized by various morphological transformations to decrease tension in the lipid bilayer. In principle, fusion and splitting are processes taking place in this case. The formation of two smaller liposomes (splitting) or one larger liposome (fusion) is the result of this transformation. The presence of a small amount of larger and smaller liposomes is demonstrated in Fig. 3. The incomplete fusion process can also lead to larger liposomes with fragments of the bilayer inside entrapped (Fig. 6C,D).

It is known that various sterols, especially cholesterol, affect structure and fluidity of phospholipid membranes. The influence of sterols like cholesterol or β-sitosterol at various concentrations up to 50 mol% on liposomal membrane fluidity, liposome size and thermal transition was tested on small unilamellar liposomes (SUV) prepared by lipid hydration methods and sonication^30,31^. The authors confirmed concentration dependent impact of sterols on membrane fluidity, but only small effect was observed with respect to the final size of SUV liposomes prepared by ultrasonication. In comparison to ultrasonication characterised by the application of high energy for disruption of large liposomes to form smaller ones, during microfluidic mixing different mechanisms are acting in liposome formation. Moreover, lower energy is used for running the process.

Microfluidic mixing as the method using low dispersive energy represents a promising technology to produce complex liposomal preparations^10^ and the application of on-chip technology as demonstrated recently^2,11^. Gadolinium complexes are used as MRI contrast agents and liposomes represents a versatile platform for the application of gadolinium *in vivo* imaging and development of theranostics^18–20^. Our manuscript describes the application of microfluidic mixing for the preparation of Ga-liposomal contrast for MRI. *In vitro* toxicological study proved potential biocompatibility as documented by Simeckova at al Gadolinium labelled nanoliposomes as the platform for MRI theranostics: *in vitro* safety study in liver cells and macrophages, in press, Scientific Reports.

## Conclusions

Lipid composition affects fluidity and elasticity of the bilayer and represents an important factor for the preparation of liposomes by microfluidic mixing. The right setting of the fluidity of lipid composition allows the preparation of liposomes with desired physical properties like size distribution, morphology. Microfluidic mixing can also be used for *in situ* preparation of therapeutics and contrast agents for diagnostics, minimizing problems with chemical (e.g. lipid oxidation and hydrolysis) and morphological instability (e.g. aggregation, fusion). These future perspectives are of interest as point-of-care personalized liposome therapeutic treatments.

A proper understanding of the physical-chemical factors and processes leading to the formation of liposomes by microfluidic techniques to which this work contribute is very important for the development of a new technological process.

In this work, the way of liposome formation by microfluidic mixing in a “herring bone” channel is presented. The fluidity of the lipid bilayer expressed as fluorescence anisotropy of the probe N,N,N-Trimethyl-4-(6-phenyl-1,3,5-hexatrien-1-yl) was found to be the basic parameter affecting the final size of formed liposomes prepared by microfluidic mixing of an ethanol solution of lipids and water phase. Both saturated and unsaturated lipids together with various content of cholesterol were used for liposome preparation and it was demonstrated, that an increase in fluidity results in a decrease of liposome size as analyzed by DLS.

We were the first who showed the impact of fluidity on the size of liposomes produced by microfluidic mixing in herring-bone like channel and who confronted experimental results with theory (Huang *et al*.)^15^. The models of liposome formation are static while the formation of disc micelles and their vesiculation is a highly dynamic process. Static models of liposomes formation do not involve the important factors such as shearing forces occurring during rapid flow mixing in the herring-bone like channel. In our case, kinetic aspects of vesicle formation were responsible for an increase of the final size of liposomes above theoretical values.

## Experimental Section

### Chemicals

Fluorescence probes: N,N,N-Trimethyl-4-(6-phenyl-1,3,5-hexatrien-1-yl) (DPH-TMA) was purchased from Sigma-Aldrich, USA.

Lipids: 1,2-distearoyl-sn-glycero-3-phosphoethanolamine-N-diethylenetriaminepentaacetic acid (18:0 PE DTPA (Gd)), 1,2-dimyristoyl-sn-glycero-3-phosphocholine (DMPC), 1-stearoyl-2-linoleoyl-sn-glycero-3-phosphocholine (SOPC) 1,2-dioleoyl-sn-glycero-3-ethylphosphocholin (EPC 95%), 1,2-distearoyl-sn-glycero-3-phosphocholine (DSPC) and cholesterol were purchased from Avanti lipids USA, with purity of 99%. All other chemicals were purchased from Sigma-Aldrich, USA.

### Microfluidic mixing

**Figure 9:**
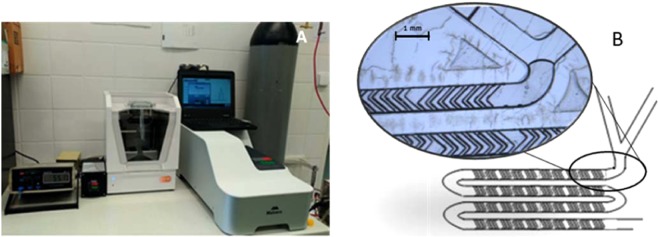
NanoAssemblr instrument settings with precise measurement of the temperature in the mixing cartridge and Zetasizer Ultra for size distribution measurement (**A**), Detail of microfluidic mixing “herring bone” structure in the NanoAssemblr Microfluidic Cartridge (**B**).

The required amount of individual lipids according to the desired composition (Table 2) were dissolved in anhydrous ethanol and in a mixture of EtOH:DMSO (1:1) for composition 2,3,4 and 1, respectively at 4 mg/ml. The organic and aqueous phase (Milli-Q water) were rapidly mixed using the NanoAssemblr Benchtop instrument (Precision NanoSystems, Canada) at defined Flow Rate Ratios (FRR) 1:3 and Total Flow Rate (TFR) 7 ml/min to form unilamellar liposomes 1 mg/ml of lipid. During the mixing process, the temperature was controlled using NanoAssemblr Benchtop Heating Controller accessory (Precision NanoSystems, Canada). For preparation of liposomes containing DSPC, the temperature was set to 65 °C, respectively. NanoAssemblr instrument setting is presented at Fig. 9.

**Table 2:** Lipid compositions of the liposome formulation.

lipid	composition (molar % of lipid)				
	1	2	3	4	5
18:0 PE DTPA (Gd)	5	—	—	—	—
DMPC	—	—	—	—	100
SOPC	—	—	—	—	100
DSPC	—	—	(90–50)	—	—
EPC	65	70	—	(100–50)	—
Cholesterol	30	30	(10–50)	(0–50)	—

### Characterization of liposomes by dynamic light scattering (DLS)

Samples of liposomes suspension (1 mg/ml and 2 mg/ml) were diluted 1:10 using Milli-Q water. The diluted suspension was placed in a disposable, low volume cuvette with path length 10 mm (Malvern Instruments, UK). For the liposomes size distribution, the Zetasizer Ultra (Malvern Panalytical, UK) operating at detection angle of 173° at room temperature was used.

### Characterization of liposomes by electron microscopy (TEM, EDX, Cryoelectron microscopy)

#### Transmission electron microscopy

Liposomes have been suspended inside a drop of PBS. The subsequent suspension got included with a grid coated with Formvar (Sigma-Aldrich, Czech Republic) and carbon (Agar Scientific, Austria). The lattice got expelled from the suspension after 1 min, and the leftover water dried with a segment of filtration paper. Philips 208 S Morgagni (FEI, Czech Republic) at 7,500× amplification and a quickening voltage of 80 kV was used for samples visualization.

#### Identification of Gd in liposomes by TEM-EDX

We used liposomes without negative staining, only labelled by gadolinium lipid. Transmission electron microscope Jeol 2100, 100 kV coupled with energy dispersed spectroscopy system (Silicon Lithium Detector, Oxford x-MAX 80 T, SSD, England) was used for chemical analysis and detection of gadolinium in liposomes.

#### Cryo-EM sample preparation and micrograph acquisition

Previously published methods were applied for sample preparation^6^.

#### Characterization of liposomes by Atomic force microscopy (AFM)^32^

This picture has been obtained by the NanoWizard 4 (JPK) instrument. The measurement was proceeded in the QI Mode in combination with qp-BioAC-10 cantilever. Liposomes were resuspended in PBS buffer and fixated on freshly cleaved mica slides.

### Measurement of anisotropy as a parameter of fluidity of liposomal membrane

Steady-state fluorescence anisotropy measurements were obtained in the L-format using Chronos DFD Fluorescence spectrometer (ISS, USA) equipped with 300 W Cermax xenon arc lamp (ISS, USA), calcite Glan-Thompson polarizer, concave holographic grating monochromator and PMT detector. Monochromator was set up at 355 nm and 430 nm for excitation and emission wavelength respectively. The correction factor of emission monochromator transmission efficiency was obtained from the ratio of emission intensity at 0° and 90° with the excitation polarizer oriented at 90°. Measurements were performed at 25 °C and 55 °C for liposomes containing EPC (composition 4) and DSPC (composition 3). Values were recorded using Vinci software (ISS, USA) in a twenty-fold repeat (Fig. 9).

## Supplementary information

Supplementary information.
