# Supplementary material for: Preparation of nanoliposomes by microfluidic mixing in herring-bone channel and the role of membrane fluidity in liposomes formation
Source: Sci Rep. 2020 Mar 27;10:5595. doi: 10.1038/s41598-020-62500-2 (PMC7101380; doi:10.1038/s41598-020-62500-2)

**Preparation of nanoliposomes by microfluidic mixing in herring-bone channel and the role of membrane fluidity in liposomes formation**

**SUPPLEMENTARY INFORMATION**

Jan Kotouček^†a^, František Hubatka^†a^ Josef Mašek^a^, Pavel Kulich^a^, Kamila Velínská^a^, Jaroslava Bezděková^a,b^, Martina Fojtíková^a^, Eliška Bartheldyová^a^, Andrea Tomečková^a^, Jana Stráská^c^, Dominik Hrebík^d^, Stuart Macaulay^e^, Irena Kratochvílová^f*^, Milan Raška^*,a,g^, and Jaroslav Turánek^*,a^

*a) Department of Pharmacology and Immunotherapy, Veterinary Research Institute, v.v.i., Hudcova 70, 621 00 Brno, Czech Republic*

*b)* *Mendel University in Brno, Department of Chemistry and Biochemistry,* *Zemedelska 1, 61300 Brno, Czech Republic*

*c) Regional Centre of Advanced Technologies and Materials, Palacký University, Šlechtitelů 11, 78371 Olomouc, Czech Republic*

*d) Central European Institute of Technology CEITEC, Structural Virology, Masaryk University, Kamenice 753/5, 62500 Brno, Czech Republic*

*e) Malvern Panalytical, Malvern, Worcestershire, United Kingdom*

*f) Institute of Physics, Czech Academy of Sciences, Prague, Czech Republic*

*g) Department of Immunology, Faculty of Medicine and Dentistry, Palacky University Olomouc, Hněvotínská 3, 775 15 Olomouc, Czech Republic*

† Contribution of two first authors is equal

* Corresponding authors:

Res. et Ass. Prof. RNDr. Jaroslav Turánek, Res. Prof.. [turanek@vri.cz](mailto:turanek@vri.cz)

Prof. MUDr. Milan Raška, Ph.D. [milan.raska@upol.cz](mailto:milan.raska@upol.cz)

Ass. Prof. Ing. Irena Kratochvílová, Ph.D. [krat@fzu.cz](mailto:krat@fzu.cz)


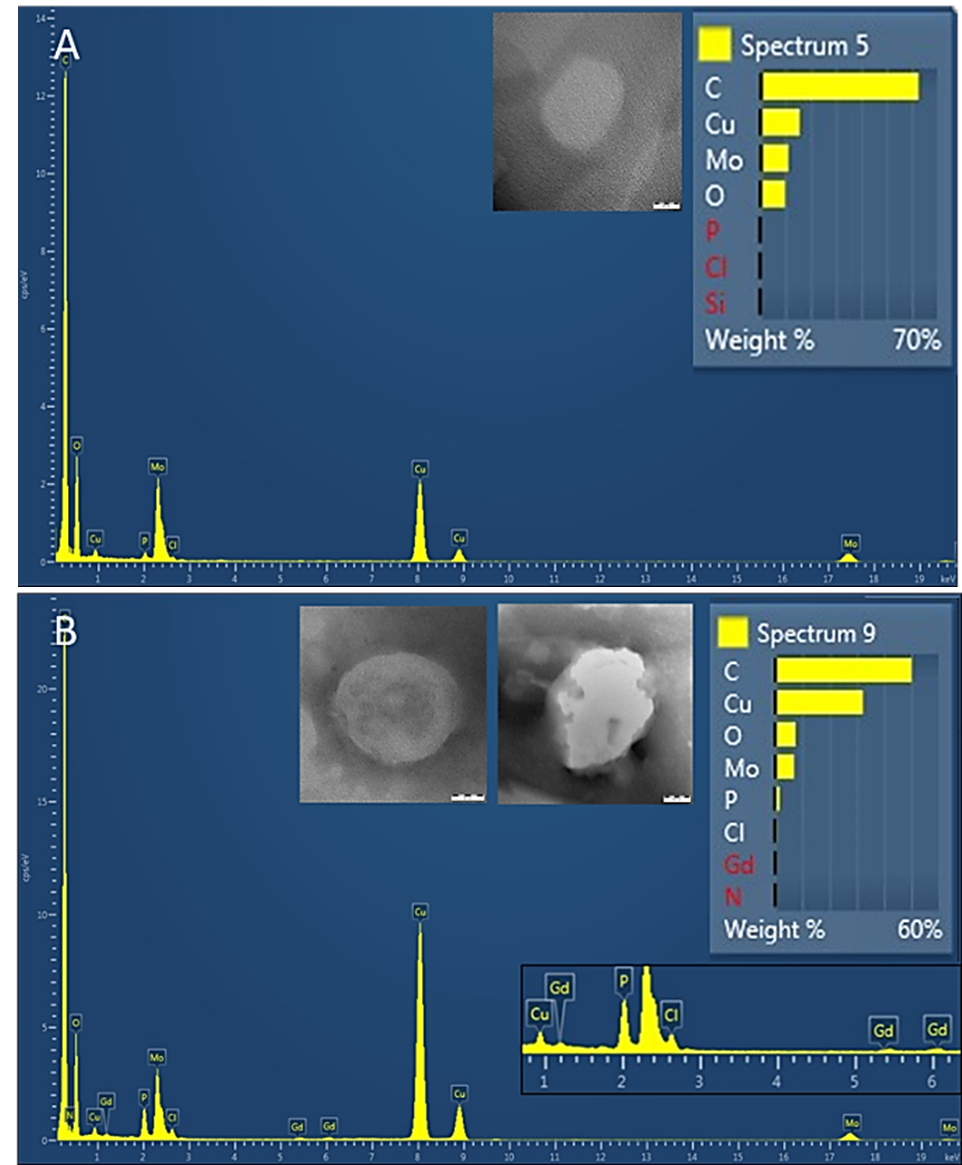


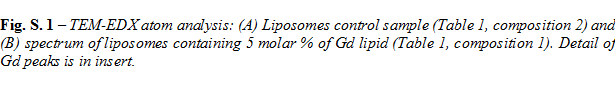

Supplement: Supplementary file 1 — Supplementary information. [file 41598_2020_62500_MOESM1_ESM.docx]
